# Supplementary material for: Integrated Analysis of the Lung Microbiome and Metabolome Reveals Associations Between Amino Acid Metabolism and Pulmonary Fibrosis in a Bleomycin-Induced Mouse Model
Source: Int J Mol Sci. 2026 Jun 30;27(13):5895. doi: 10.3390/ijms27135895 (PMC13362081; doi:10.3390/ijms27135895)
Supplement: Supplementary file 1 [file ijms-27-05895-s001.zip › result/5.MetKeggEnrichment/B_14d.vs.C_14d/B_14d.vs.C_14d_all.KEGG_Enrich.scatterplot.pdf]

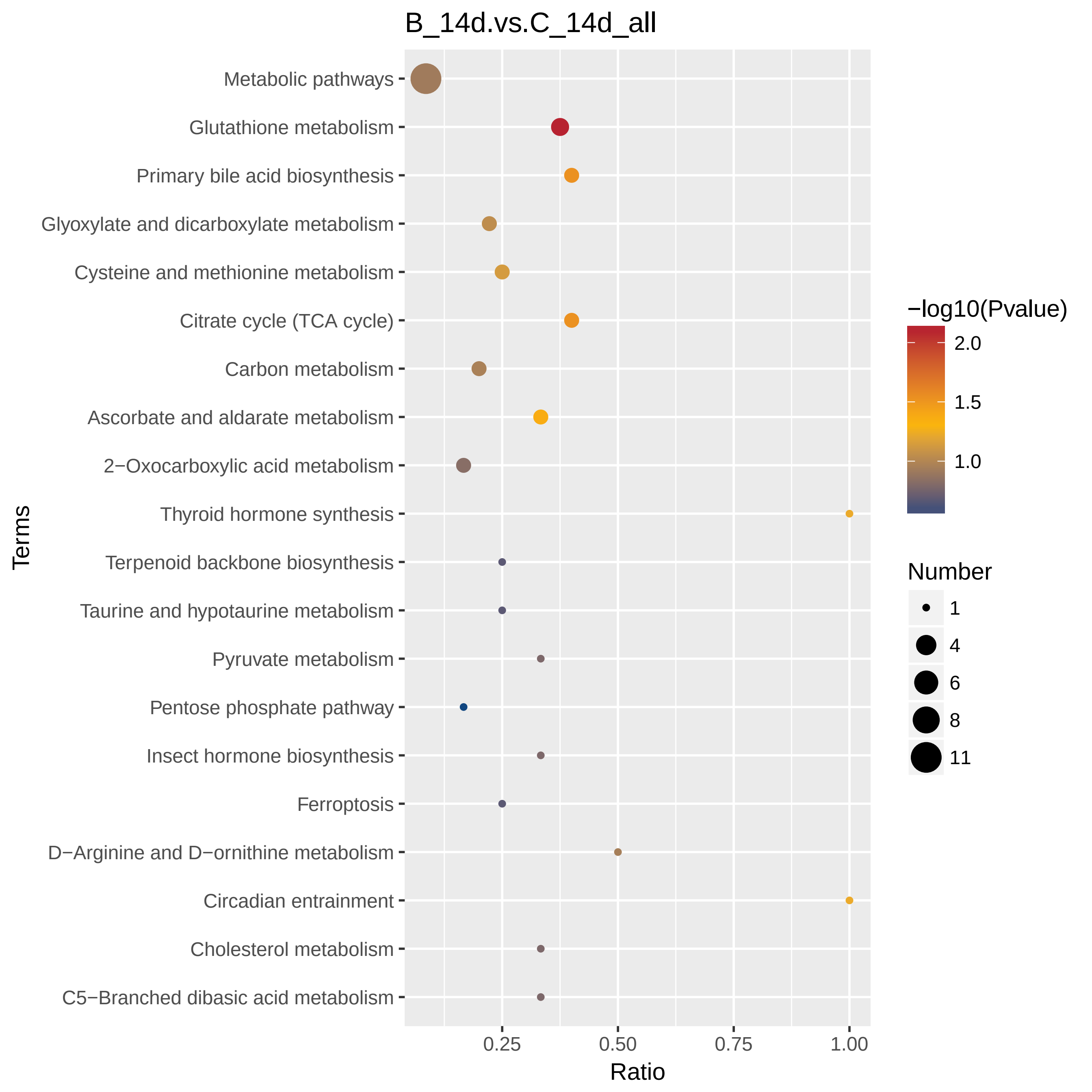
B_14d.vs.C_14d_all
Metabolic pathways
Glutathione metabolism
Primary bile acid biosynthesis
Glyoxylate and dicarboxylate metabolism
Cysteine and methionine metabolism
Citrate cycle (TCA cycle)
Carbon metabolism
Ascorbate and aldarate metabolism
2−Oxocarboxylic acid metabolism
Thyroid hormone synthesis
Terpenoid backbone biosynthesis
Taurine and hypotaurine metabolism
Pyruvate metabolism
Pentose phosphate pathway
Insect hormone biosynthesis
Ferroptosis
D−Arginine and D−ornithine metabolism
Circadian entrainment
Cholesterol metabolism
C5−Branched dibasic acid metabolism
Terms
0.25
0.50
0.75
1.00
Ratio
−log10(Pvalue)
2.0
1.5
1.0
Number
1
4
6
8
11
